# Field trial of efficacy of the Leish-tec^®^ vaccine against canine leishmaniasis caused by *Leishmania infantum* in an endemic area with high transmission rates

**DOI:** 10.1371/journal.pone.0185438

**Published:** 2017-09-27

**Authors:** Gabriel Grimaldi, Antonio Teva, Claudiney B. dos-Santos, Fernanda Nunes Santos, Israel de-Souza Pinto, Blima Fux, Gustavo Rocha Leite, Aloísio Falqueto

**Affiliations:** 1 Instituto Gonçalo Moniz, Fiocruz, Salvador, Brazil, Brazil; 2 Escola Nacional de Saúde Pública, Fiocruz, Rio de Janeiro, Rio de Janeiro, Brazil; 3 Centro de Ciências da Saúde, Universidade Federal do Espírito Santos, Vitória, Espírito Santo, Brazil; Academic Medical Centre, NETHERLANDS

## Abstract

**Background:**

Because domestic dogs are reservoir hosts for visceral leishmaniasis (VL) in Brazil, one of the approaches used to reduce human disease incidence is to cull infected dogs. However, the results of controlled intervention trials based on serological screening of dogs and killing of seropositive animals are equivocal. A prophylactic vaccine to protect dogs from being infectious to the sand fly vector could be an effective strategy to provide sustained control. Here, we investigated whether a currently licensed commercial subunit rA2 protein–saponin vaccine (Leish-tec^®^) had an additional effect to dog culling on reducing the canine infectious populations.

**Methodology/Principal findings:**

This prospective study was conducted in an *L*. *infantum* highly endemic area of southeast Brazil. At the onset of the intervention, all of the eligible dogs received through subcutaneous route a three-dose vaccine course at 21-day intervals and a booster on month 12. For the purpose of comparison, newly recruited healthy dogs were included as the exposed control group. To ascertain vaccine-induced protection, dogs were screened on clinical and serological criteria every 6 months for a 2-year follow-up period. Antibody-based tests and histopathological examination of post-mortem tissue specimens from euthanized animals were used as a marker of infection. The standardized vaccine regime, apart from being safe, was immunogenic as immunized animals responded with a pronounced production of anti-A2-specific IgG antibodies. It should be noted the mean seroconversion time for infection obtained among immunized exposed dogs (~ 18 months), which was twice as high as that for unvaccinated ones (~ 9 months). After two transmission cycles completed, the cumulative incidence of infection did differ significantly (*P* = 0.016) between the vaccinated (27%) and unvaccinated (42%) dogs. However, the expected efficacy for the vaccine in inducing clinical protection was not evident since 43% of vaccine recipients developed disease over time. Our estimates also indicated that immunoprophylaxis by Leish-tec^®^ vaccine in addition to dog culling might not have an impact on bringing down the incidence of canine infection with *L*. *infantum* in areas of high transmission rates.

**Conclusions/Significance:**

Leish-tec^®^ as a prophylactic vaccine showed promise but needs to be further optimized to be effective in dogs under field conditions, and thereby positively impacts human incidence.

## Introduction

Visceral leishmaniasis (VL; also known as kala-azar) is a severe vector-borne disease, which if left untreated is almost always fatal. Approximately 0.2 to 0.4 million new human VL cases occur annually worldwide but the disease is still grossly underreported [1]. Anthroponotic VL occurs in regions where *Leishmania donovani* infections are endemic (South Asia, East Africa, and parts of the Middle East), whereas zoonotic VL is found in areas of *Leishmania infantum* (syn. *L*. *chagasi*) transmission (Latin America, southern Europe, North Africa, and West and Central Asia) [1,2]. Molecular characterization of New World *L*. *infantum* revealed low genetic heterogeneity among populations and its recent Old World origin [3].

In the Neotropics, *L*. *infantum* is usually transmitted by *Lutzomyia longipalpis*, and the population density of these insects in the peridomestic setting can reach very high levels [4]. The essential maintenance cycle of the parasite involves foxes (*Dusicyon vetulus* and *Cerdocyon thous*) [5, 6] and opossums (*Didelphis Albiventris* and *D*. *marsupialis*), which can be naturally infected [7, 8], though their epidemiologic role as sylvatic or peridomestic reservoir hosts remains unknown. In VL-endemic regions of the Mediterranean and Latin America, the domestic dog (*Canis familiaris*) is the main parasitic reservoir host of human infection [9, 10]. In areas of high transmission, a high prevalence of canine infection is associated with high-risk of human disease [11].

Ample evidence supports the notion that host genetic variability and specific immune responses influence the outcome of *L*. *infantum* infection in dogs [9]. Whether or not strain variants of this parasite [3] may be equally influential in the development of the disease phenotype is still to be determined. Canine leishmaniasis (CanL) caused by *L*. *infantum* appears as chronic wasting systemic disease characterized by skin lesions (in which parasites can be detected), lymphadenopathy, ocular lesions, enlarged spleen, nose bleeding (epistaxis),abnormal nails (onychogryphosis), hematuria, anemia, progressive muscular atrophy, joint and bone lesions, and cachexia[12].

Similar to the situation in human VL, progressive disease in dogs is associated with an increasing state of immunosuppression, attributed to the presence of immunoregulatory cytokines, notably interleukin (IL)-10 [9,13]. Disease exacerbation is also associated with pronounced increases in parasite-specific antibody titers and the strength of the dog’s anti-*Leishmania* antibody response has been shown to correlate with its clinical and parasitological statuses [10, 14]. Severely affected dogs do not survive the disease, but subclinical infection is quite common in dogs [9, 15]. Both diseased and sub-clinically infected dogs might be considered as a source of the parasite to the sand fly vector but the probability of *L*. *longipalpis* becoming infected from an infected dog appears to be higher in cases of clinical disease [16].

Measures used to control zoonotic VL such as mass detection of seropositive dogs followed by culling and the use of residual insecticide spraying of houses and animal shelters are not always possible [17] and showed controversial results [18]. Treatment of infected animals could reduce the canine reservoir, and thereby positively impacts human incidence. However, drug-cured dogs often relapse and may remain infectious to sand flies [19, 20]. Moreover, chemotherapy is not widely recommended [2] since both human and canine treatment are performed with the same drugs, thus raising the risk of emergence of drug-resistant parasites. Although the use of topical insecticide treatment (collars, spot-on devices and sprays) can reduce the risk of contracting VL [21, 22], it is costly and difficult to implement at a national level [23]. Nevertheless, most experts believe that prophylactic or possibly post-exposure vaccination will be essential for ultimate control of the disease [24–27].

Currently, there is only one commercially available canine vaccine (Leish-tec^®^) and new vaccines under development include recombinant antigen vaccines and both live and killed whole-cell vaccines [25]. Field trials have shown promise in both prophylactic and therapeutic approaches in CanL [24,26,27], though evaluation of vaccine potential has been hampered by the difficulty to accurately measure immune responses, particularly when discriminating vaccine- from infection-induced antibody responses by the serological tests [24,28]. This is an important issue when managing vaccine recipients since immunized animals becoming seropositive can be unnecessarily euthanized.

Preliminary trials on animal models with the amastigote-specific *L*. *donovani* A2 cysteine proteinase [29] have shown some levels of protection following delivery by numerous immunization regimens [30–32] and under the product name Leish-tec^®^ has also been promising in naturally exposed dogs [28], but larger studies are required to confirm if canine prophylaxis with this licensed anti-*Leishmania* vaccine product could reduce transmission and prevent disease. On the basis of these observations, we conducted a prospective intervention study to determine whether Leish-tec^®^ vaccination as an adjunct to the culling of potentially infectious (rather than simply infected) dogs would have an impact on the prevalence and incidence of canine infection in an endemic area with high *L*. *infantum* transmission rates. The objective was to provide support for an integrated vaccine and culling strategy capable to reduce this reservoir of infection and thereby breaking the transmission to humans.

## Methods

### Ethics statement

Enrollment of all dogs in this study was performed with the owner’s consent who was informed about the risk of the procedures and the requirement for a 2-year follow-up. This research complied with all relevant national (CONCEA: Brazilian Government Council for Control of Animal Experimentation) and international guidelines for care and use of animals in research. In accordance with the Brazilian National Health Foundation [17], seropositive dogs developing clinical disease were submitted to euthanasia with a lethal overdose of thiopental sodium (Euthasol, Virbac Animal Health, Forth Worth, TX) administered intravenously. The dog culling was justified for many reasons connected with health, the environment, and conservation [2]. The protocol was approved by the Committee on the Ethics of Animal Experiments of the Centro Universitário do Espírito Santo (CEUA-UNESC), under Permit Number: 199296/2013.

### Study location

The study was conducted in an *L*. *infantum* highly endemic area of southeast Brazil (Pancas, Espírito Santo State). In a previous survey conducted in 2003 [15], 42% of the human residents were leishmanin-positive reactors and 47% were seropositive by ELISA assays based on crude and recombinant leishmanial antigens, whereas nearly 57% of the indigenous dogs were also seropositive to the same antigens. During the surveys, *L*. *longipalpis* was the most commonly found sand fly species (80% of the collected samples) inside houses and in the peridomestic setting. The lowest incidence of canine infection (9%) in this area occurred in October 2010 following the culling of potentially infectious dogs but rising again to 36% during the 15-month period after intervention [33]. At that time, there were many more cases of the clinical disease in dogs than in people.

### Animals and study design

As in other areas of Brazil in which VL is endemic [18] and as recommended by WHO [2], the epidemiological control in Pancas emphasizes the serological surveillance of dogs, and the elimination of seropositive animals [17]. However, the culling program has been ineffective in the study area, probably due to the low sensitivity of diagnostic methods and delay in the removal of infectious dogs [10]. As the presence of circulating antibodies against the recombinant (r) K26, K28, and K39 antigens of *L*. *infantum* reflects infection [15,33–35], we used serological testing based on the Dual-Path Platform (DPP) rK28 fusion protein chromatographic immunoassay for this dog screening-and-culling intervention trial. The rK28-based DPP^®^ CanL rapid test (Bio-Manguinhos/Fiocruz, Rio de Janeiro, Brazil) was designed to detect antibodies against the rK9/K26/K39 antigens of *L*. *infantum*. It has been reported [34,35] to be as sensitive as rK26- or rK39-based enzyme-linked immunosorbent assay (ELISA) and superior to immunofluorescence assays in detecting clinically symptomatic and asymptomatic canine carriers of *L*. *infantum*. In addition, it has been recently approved for dog screening by the Brazilian Public Health authorities.

The initial serological screening disclosed by this ready-to-use disposable device (which requires a 5 μL serum or whole fresh blood sample) excluded all dogs with positive serology for K28-specific antibody reactivity. In accordance with the Ministry of Health policy [17], the local public health service personnel removed all infected animals to a veterinary public health post where they were eliminated. The remaining healthy K28-seronegative dogs were admitted to the study. For the purpose of comparison, introduced sentinel beagles (purchased from a dog supply company located in a VL-free area in Brazil) and newly recruited healthy dogs (added as replacements during the study period) were included as the exposed control group. The indigenous animals enrolled in this study were companion animals (composed mainly of guard dogs, hunting dogs, and pet dogs) of mixed breed (males and females, with a mean age of 2.6 years).

Since the heterogeneity of disease transmission within the study area [15] could generate imbalances in the baseline comparisons among canine groups, control animals were domiciled in residences near the vaccine recipients, thus ensuring equal risk of natural infection. The studied animals received no additional protection or treatment in the care of their owners other than standard clinical care and immunizations. It should be noted that no human VL case was identified by clinical examination or vector control program occurred within the study period.

### Vaccination protocol and follow-up serological evaluations

Leish-tec^®^ vaccine comes in ready-to-use flasks containing 100 μg of rA2 protein and 500μg of saponin (Riedel) in 0.9% of saline solution (1.0 ml). All vaccine doses from lot 004/13 were maintained at 4°C until use. The vaccination was performed in three subcutaneous injections on the back at dose intervals of 21 days, accordingly to the manufacturer’s instructions. The surviving dogs received a boosting injection 1 year after the first vaccine course for the recall of protective immune responses [36]. Leish-tec^®^ has been shown to be well-tolerated by canines but vaccine-induced transient adverse reactions may occur [28]. Therefore, the injected dogs were particularly monitored by veterinarians for 72 hours after administration of each vaccine dose for safety evaluation. After this period, each dog owner was instructed to contact veterinarians if the animals presented any side effect. In safety evaluation, the dogs were physically examined and the site of vaccine injection was checked and the types of local and/or systemic adverse reactions were recorded.

Blood collected by venipuncture from selected dogs prior to vaccination and at 1, 6, 12, 18 and 24 months post-complete vaccination was centrifuged and stored in aliquots at– 20°C. Serum samples were tested for antibodies using the A2- or*-Leishmania* promastigote antigen (LPA)-based ELISA technique. Anti-A2 serological responses were measured as previously described [36]. Briefly, 96-well plates were sensitized overnight with 250 ng/well rA2 in carbonate buffer. Sera were assayed at the dilution of 1:100, and the peroxidase-labeled antibodies specific to canine IgG, IgG1, or IgG2 isotypes (Sigma) were used at the 1:25,000, 1:15,000, or 1:35,000 dilution, respectively. The cut-off values (cut-off = mean OD + 3 x SD) were determined with sera from healthy dogs born in a VL-free area of Brazil. We used LPA-sensitized ELISA plates for the detection of total IgG anti- *Leishmania* antibodies according to the manufacturer’s instructions (Bio-Manguinhos/Fiocruz, R. Janeiro, Brazil). Positive and negative control sera were included in each assay run. To perform a comparative analysis of the OD values obtained in different plates, an inter-plate correction factor was determined as described by Fernandes et al. [28].

### Assessment of *L*. *infantum* infection and disease development

Clinical signs of VL can take months to develop after infection, and may spontaneously resolve. Thus in order to distinguish between healthy and sick dogs, animals enrolled was examined by specific serology, clinical evaluation and/or histopathological analysis on day 0 and at various time points after initiating the vaccination. On the basis of both the number K28-specific antibody units and their changes over time, we were able to reliably identify dogs from both vaccinated (S1 Table) and control (S2 Table) groups that were potentially noninfectious and infectious. Clinical assessment was performed by veterinary practitioners under generally recognized standards of care and medical practice. All seropositive dogs underwent gross physical examinations for the appearance of typical signs of CanL (such as alopecia, exfoliative dermatitis, ulcerative skin lesions, conjunctivitis, lymphadenopathy, onycogryphosis, weight loss, apathy, anorexia and renal failure). Animals were further classified as sub-clinically infected, when no suggestive signs of the disease were detected or sick when at least one characteristic clinical sign was observed.

In order to limit the transmission, any dog considered potentially infectious to sand flies [i.e., cases with a DPP-determined K28-specific antibody reactivity of ≥ 15 relative light units (RLU) and/or suggestive signs of the disease] was removed from the area within 8 days after being diagnosed, euthanized, and when was possible, submitted to necropsy. For assessment of parasites, post-mortem tissue specimens were removed from ear skin, skin-draining lymph node, liver and spleen and processed for histopathological examination. Dogs with antibody levels lower than 15 RLU continued in follow-up to assess the infection behaviour in the local canine population (they were sampled at bi-monthly intervals, and euthanized any time in the event of developing progressive infection); their survival was also monitored by contacting individual owners monthly during the study.

### Dog surveys

The house-to-house surveys ran from April 2013 to April 2015, during which time all dogs were screened on clinical and serological criteria. As dogs enrolled were not withdrawn from the endemic area, each animal received a microchip for identification. Sampling was conducted continuously over each 6-month sampling period. Data were analyzed at the end of each sampling period and again at the completion of all sampling periods. The first cohort contained 202 dogs, and additional animals were enrolled into the study at sampling dates.

### Statistical analysis

We used the One-Way ANOVA analysis (IBM^®^ SPSS^®^ Statistics Version 20) with Dunnett’s Multiple Comparison tests for comparative analysis of quantitative data. Means were defined as significantly different when *p*-value < 0.05. Changes in prevalence and incidence of canine infection during the study period were compared using the χ^2^ test and the χ^2^ test for trend over time.

## Results

### Safety evaluation

The vaccine at the doses employed was safe and well-tolerated by the animals. Only a mild painful swelling reaction was seen at the site of injection and general adverse reactions (such as claudication, apathy, and anorexia) were observed, more often after the second or third vaccine dose, in 11% (17 of 151) of the cases. The side effects were transient, lasting up to four days.

### A2 specific antibody responses in dogs

**Fig 1:**
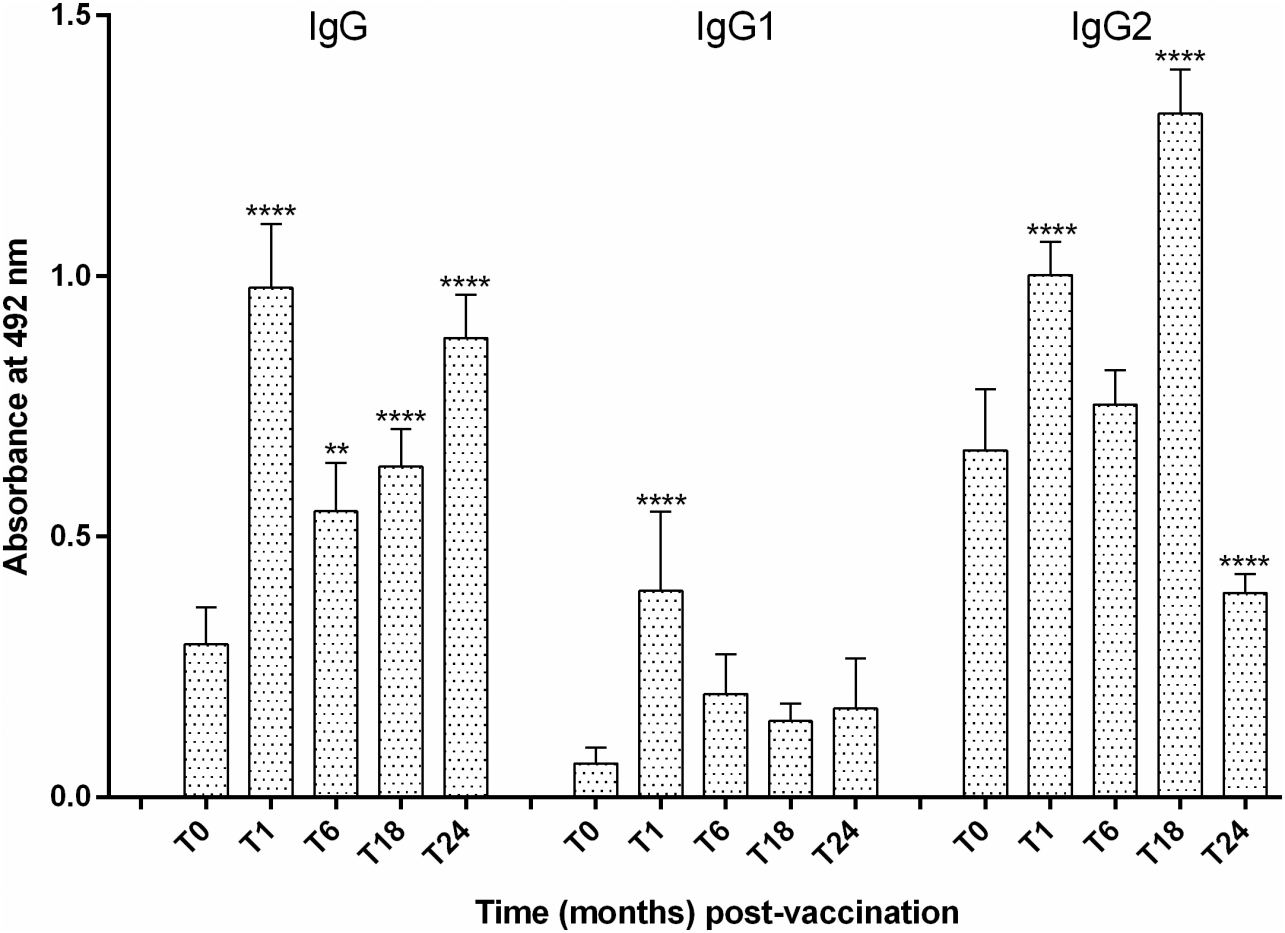
Antibody responses to A2 in dogs vaccinated with Leish-tec^®^ and exposed to natural *Leishmania infantum* infection. Levels of anti-A2 IgG, IgG1, and IgG2 antibodies in pre-immune sera and at different time points after vaccination were assessed by ELISA. Plates were coated with 250 ng/well of rA2 and using a single serum dilution of 1:100. The lower limit of positivity (cut-off) was determined for each ELISA plate by using the mean plus 3 standard deviations of the A492 values for 5 normal controls. Significant differences between pre- and post-vaccination mean values of optical density at 492 nm are indicated as **(*p*-value < 0.01) or **** (*p*-value < 0.001).

**Fig 2:**
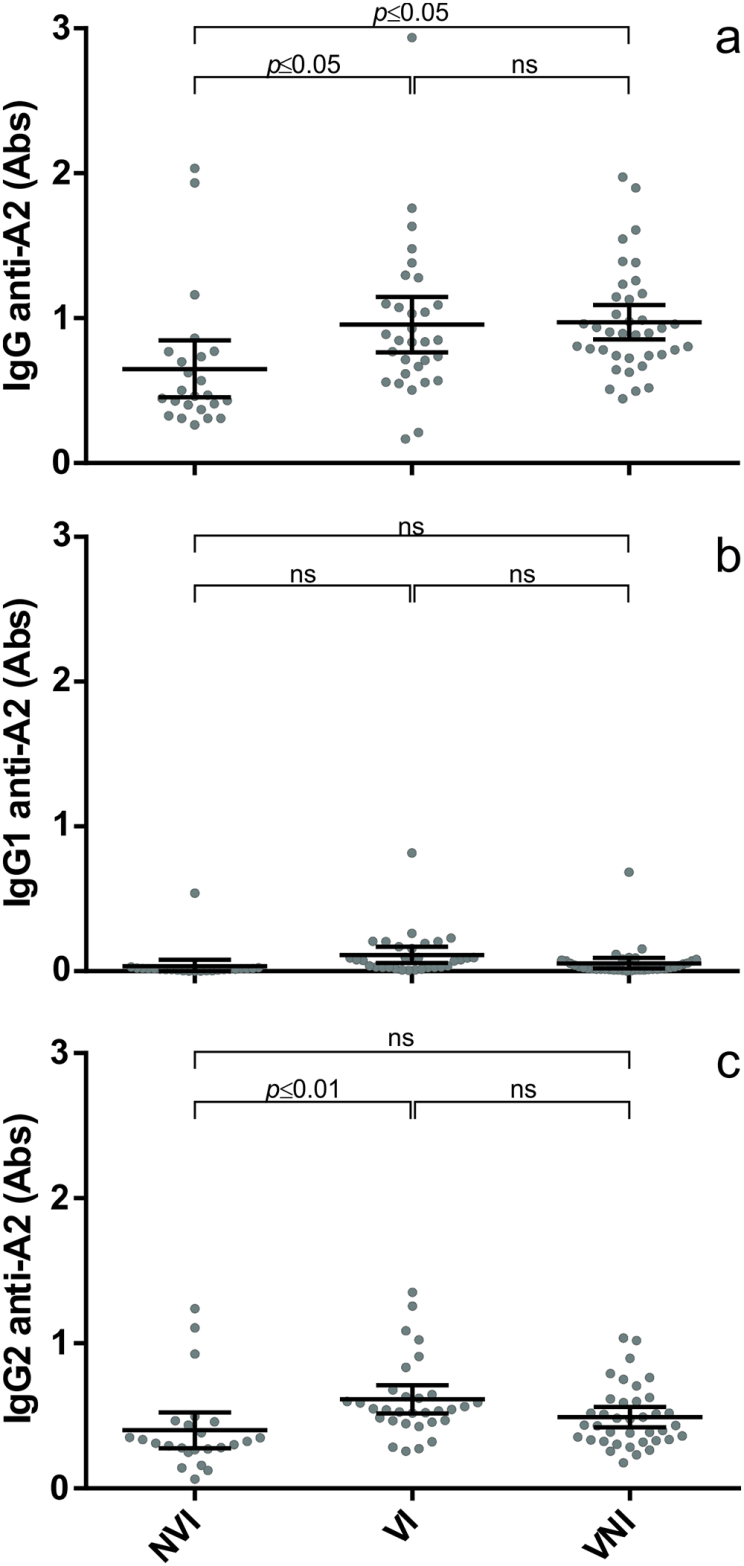
Comparative levels of circulating anti-A2 IgG (panel a), IgG1 (panel b), and IgG2 (panel c) antibodies in dogs from different groups (NVI, non-vaccinated infected; VI, vaccinated infected; VNI, vaccinated non-infected). Anti-A2 serological responses were measured as described in Fig 1. The bars indicate significant differences in mean values of OD at 492 nm between animal groups.

Fig 1 shows the change over time in anti-A2 IgG, IgG1 and IgG2 antibody titers in vaccine recipients. Of note, the high proportion of tested dogs (79%; 27/34) that displayed positive anti-A2 antibody titers prior to vaccination, thus revealing that may have been infected during the 2–3 weeks lapse between the screening and the first vaccine injection. Nonetheless, all tested vaccine recipients (*n* = 62) responded with increased production of anti-A2 total IgG and specific IgG1and IgG2 antibodies 1 month after vaccination. Of note, the levels of anti-A2 IgG2, but not IgG1, antibodies significantly increased in vaccinated dogs throughout the tests. As can be seen in Fig 2, our estimates also showed significant differences in mean absorbance values relating to IgG and IgG2 anti-A2 antibody responses between vaccine recipients and control animals that became infected with time, thus confirming the immunogenicity of the rA2 antigen.

### Protection of infection and prevention of disease progression

In previous reports [33, 34], we have convincingly shown that the dog’s K28-specific antibody response correlates with its clinical and parasitological statuses. Prior to immunization, all animals included in the trial were apparently healthy and K28-seronegative. Positive DPP signals (mean ± SEM) were very variable in dogs possibly being affected either by sub-patent (7.88 ± 1.29) or patent (35.22 ± 2.05) infection and significantly higher (F = 269.53, p <0.001) in sick animals (83.15 ± 4.18). In the agreement, the antibody levels disclosed by the LPA-based ELISA were remarkably higher in dogs suffering from a symptomatic patent infection.

As can be seen in S3 Table, 40 of 151 vaccines recipients and 33 of 78 sentinels dogs surveyed during the 24-month follow-up period displayed positive serum antibody reactivity to the K28 fusion protein of *L*. *infantum*. After initial exposure, the mean seroconversion time for infection obtained among vaccinated dogs (~ 18 months) was twice as high as that for unvaccinated ones (~ 9 months). After two transmission cycles completed, there were statistically significant differences (χ^2^ = 5.768; *P* = 0.016) in incidence of infection between the vaccinated (27%; 40/151) and unvaccinated (42%; 33/78) dogs. However, by the end of the 24-month study, 62 vaccinated dogs were still alive and 19 of them (31%) had converted to a seropositive status after five consecutive negative readings (S1 Table). Noteworthy, vaccination failed at preventing disease development in susceptible hosts as the proportion of diseased dogs displaying a k28-specific increased response was 2-fold higher in the vaccinated group (44%; 18 of 40) than in control group (21.2%; 7 of 33).

**Fig 3:**
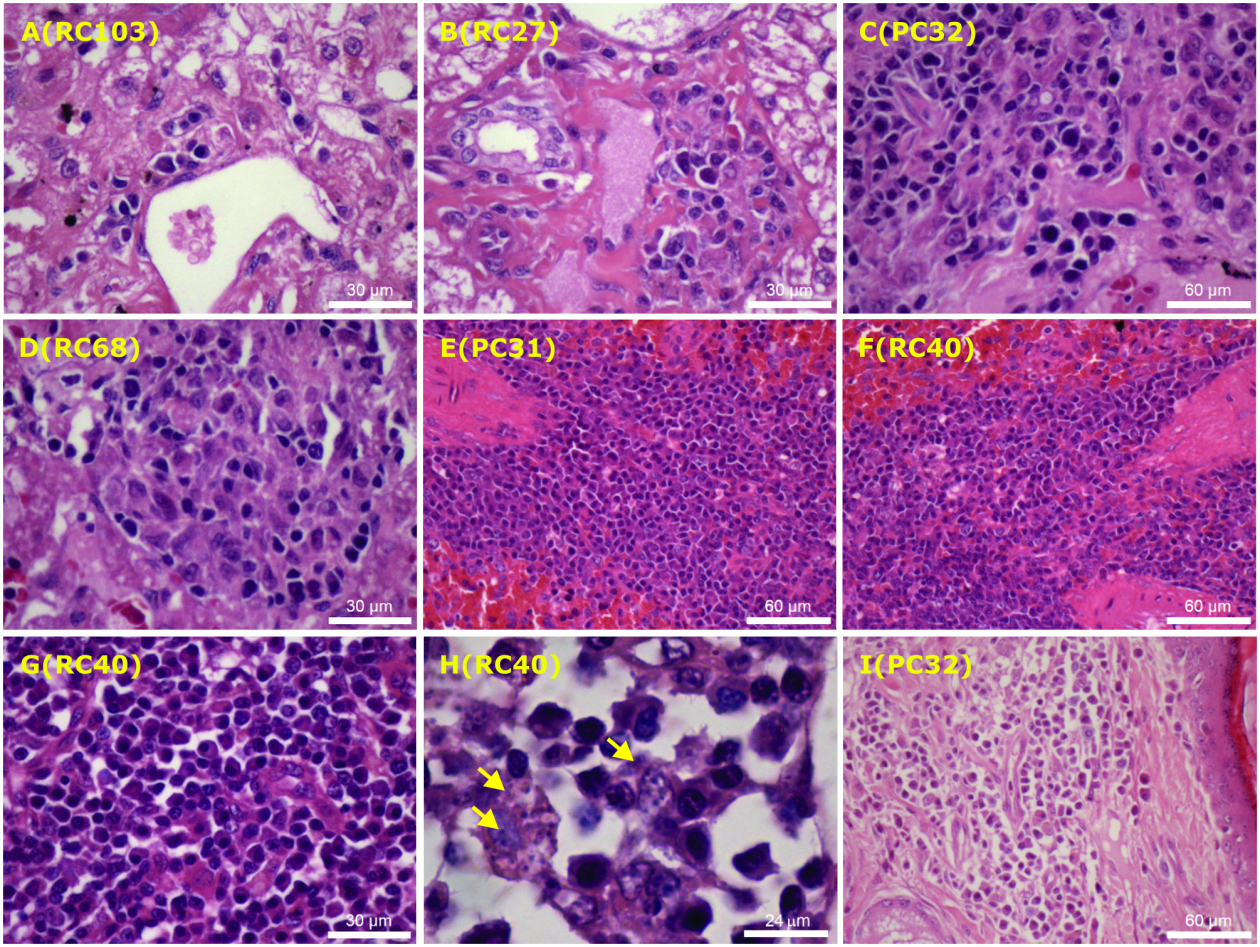
Histopathological changes in affected tissues of control (images A, C, E, I) and vaccinated (images B, D, F-H) dogs naturally infected with *Leishmania infantum*. Photomicrographs sections from the liver of dogs with subclinical infections showing minimal mononuclear cell infiltration in portal spaces (A-B), as well as intralobular granulomas containing activated but parasite-free macrophages, surrounded by plasma cells and occasional lymphocytes (C-D). Also illustrated are photomicrographs of sections from spleen (E-G), lymph node ((H) and ear skin (I) showing a more marked mononuclear cell infiltration, particularly composed of plasma cells, activated macrophages, including parasite-containing phagocytes (arrows), thus confirming the sustained course of infection. The number within parenthesis indicates dog code. Images were obtained from paraffin-embedded sections stained with haematoxylin-eosin at different magnification.

Histological examination oriented towards the detection of the parasites in post-mortem tissue specimens confirmed the presence of leishmanial infection in most necropsy cases. As can be seen in Fig 3, a clear separation between latent and active infections in canines was possible based on the histopathological findings in their affected tissues (liver, spleen, lymph node and ear skin). Whereas indistinctly delimited and more or less differentiated macrophage accumulations surrounded by few plasma cells and lymphocytes in the portal zone (A-B) was found in sub-clinically infected dogs, intra-lobular granulomas composed of concentric layers of macrophages, epithelioid cells and lymphocytes (C-D) were documented only in dogs with the active disease. Moreover, more extensive pathology found in their spleen (E-G), skin-draining lymph node (H) and ear-skin (I) included an aggregation of activated macrophages associated with the presence of parasitized vacuolated phagocytes (arrows) and a more marked plasma cell infiltration, which was consistent with the robust specific antibody responses developed by sick dogs. It is worth mentioning that no remarkable differences on pathological changes were seen when comparing vaccinated and control animals.

### Prevalence and incidence of canine infection

As summarized in Table 1, of 202 dogs surveyed in April 2013, 49 (24%) exhibited a positive DPP-determined K28-specific antibody reactivity. All (21) dogs with clinical signs of CanL were seropositive. In contrast, of the 181 apparently healthy dogs, only 28 (16%) were seropositive. Of these 49 infected dogs, 38 (78%) were euthanized (27 cases suffering from clinically symptomatic or asymptomatic patent infection), died of other causes (4) or relocated by their owners (7). The remaining seropositive animals (11 cases affected by sub-patent infection, all displaying antibody levels lower than 15 RLU) continued in follow-up to assess the infection behaviour after vaccination. By October 2013, 13 (9%) of the 153 seronegative dogs examined in April 2013 had converted to a seropositive status, 91 remained seronegative, and 49 could not be located. At that time, 28 dogs were seropositive, including 12 newly recruited ones, and 129 were seronegative, including 30 newly recruited ones. Thus, at the 6-month time point, the incidence was 13%; this rate was estimated by dividing the number of converters to seropositive status (13) by the number of dogs (both seronegative and seropositive) available for the survey (104).

**Table 1:** Summary of canine surveys in the intervention area of Pancas, Espírito Santo, Brazil in 2013–2015.

Outcome of the follow-up survey following vaccination					
Sampling interval	No. (%)	Sero-positive	Sero-negative	Removed (euthanized)	Died or moved
April 2013 –October 2013					
Positive	49 (24)	3	8	27	11
Negative	153 (76)	13	91	-	49
Total	202	16†	99‡	-	-
October 2013 –April 2014					
Positive	28 (18)	6	2	3	17
Negative	129 (82)	9	100	-	20
Total	157	15†	102‡	-	-
April 2014 –October 2014					
Positive	18 (12)	1	2	11	4
Negative	129 (88)	17	87	-	25
Total	147	18†	89‡	-	-
October 2014 –April 2015					
Positive	22 (17)	4	1	12	5
Negative	111 (83)	30	65	-	16
Total	133	34†	66‡	-	-
April 2015					
Positive	39 (35)	-	-	-	-
Negative	73 (65)	-	-	-	-
Total	112	-	-	-	-

Positive equals the total number of seropositive dogs in the follow-up group (†) plus new dogs that moved into the area that were seropositive.

Negative equals the total number of seronegative dogs in the follow-up group (‡) plus new dogs that moved into the area that were seronegative.

In April 2014, 157 dogs were surveyed and 28 (18%) of them were seropositive [15 of 144 (10%) sub-clinically infected and 13 of 13 (100%) disease]. Of these 28 infected dogs, 20 (71%) were euthanized (3), died of other causes (3) or could not be located (14). At that time, 9 (7%) of the 129 seronegative dogs converted to a seropositive status, 100 remained seronegative, and 20 could not be located. Eighteen dogs were seropositive, including 3 newly recruited ones. One hundred twenty-nine were seronegative, including 27 newly recruited ones. At the 12-month period, the incidence was 8% (9 of 109).

In October 2014, 147 dogs were surveyed and 18 (12%) of them were seropositive [13 of 142 (9%) sub-clinically infected and 5 of 5 (100%) disease]. Of these 18 infected dogs, 15 (83%) were euthanized (11), died of other causes (1) or could not be located (3). At that time, 17 (13%) of the 129 seronegative dogs converted to a seropositive status, 87 remained seronegative, and 25 could not be located. Twenty-two dogs were seropositive, including 4 newly recruited ones, and 111 were seronegative, including 22 newly recruited ones. At the 18-month period, the incidence was 16% (17 of 104).

In April 2015, 133 dogs were surveyed and 22 (17%) were seropositive [12 of 123 (10%) sub-clinically infected and 10 of 10 (100%) disease]. Of these 22 infected dogs, 17 (77%) were euthanized (12), died of other causes (2) or could not be located (3). At that time, 30 (27%) of the 111 seronegative dogs converted to a seropositive status, 65 remained seronegative, and 16 could not be located. Thirty-nine dogs were seropositive, including 5 newly recruited ones, and 73 were seronegative, including 7 newly recruited ones. At the 24-month period, the incidence was 32% (30 of 95).

**Fig 4:**
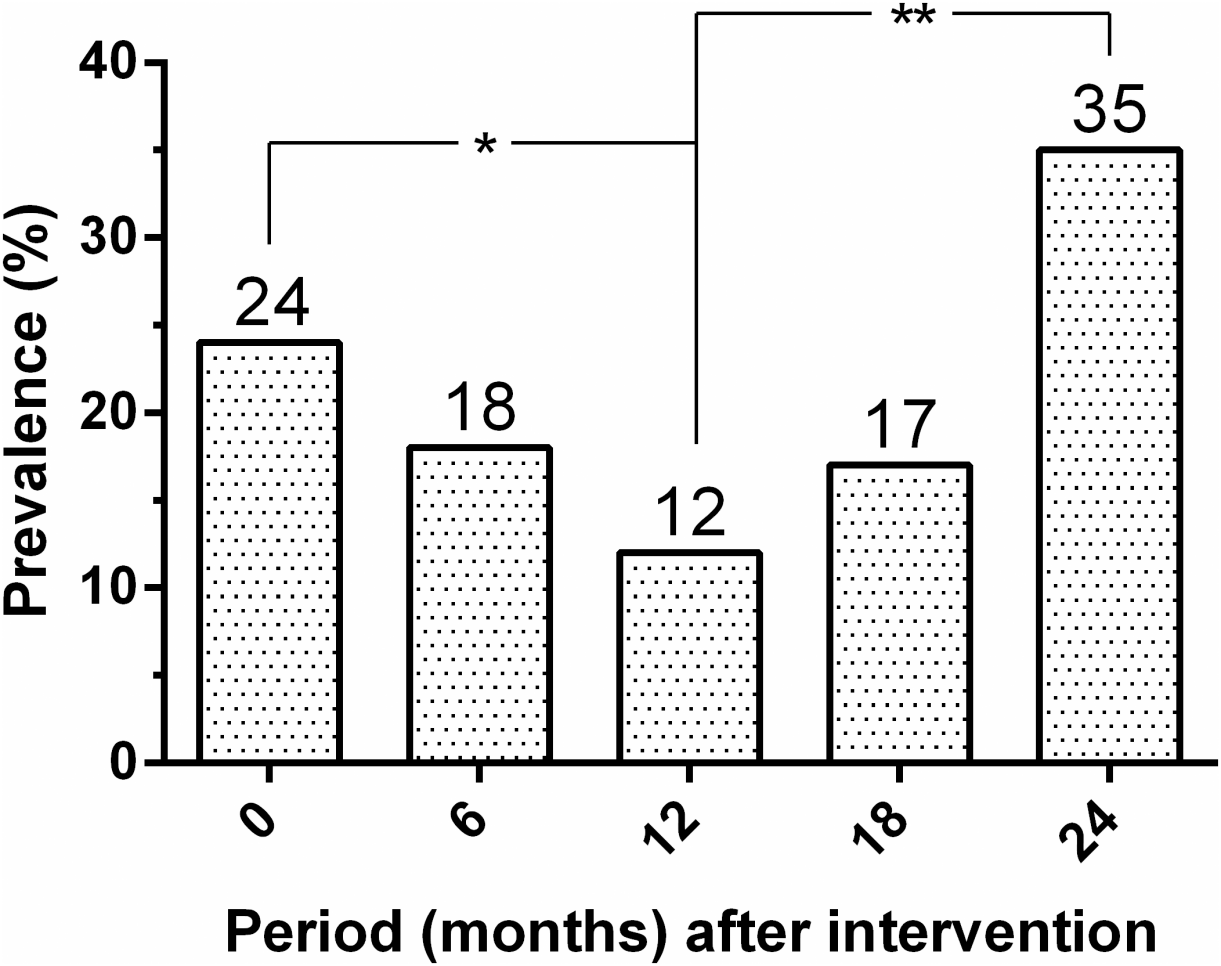
Initial seroprevalence and seropositivity rates of canine *Leishmania infantum* infection in the intervention area (Pancas, ES, Brazil, 2013–2015). Significant differences between seropositive rates over time are indicated as *(P = 0.006) or ** (P < 0.001).

**Fig 5:**
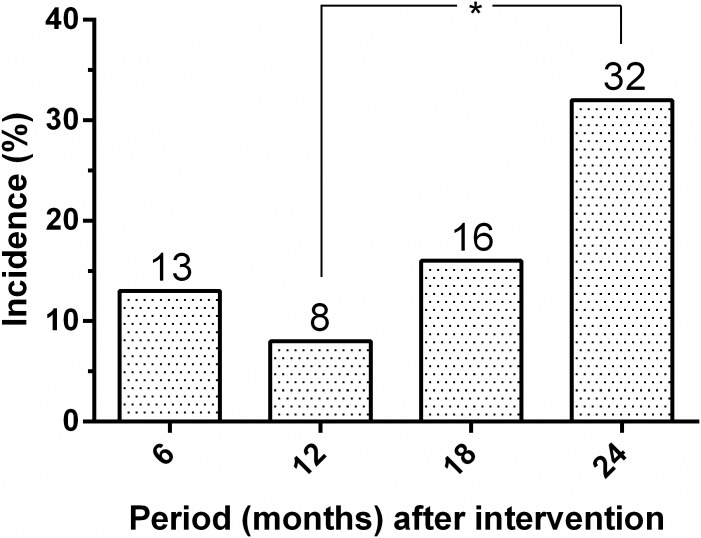
Cumulative incidence of seroconversion for canine *Leishmania infantum* infection in the intervention area (Pancas, ES, Brazil, 2013–2015). *Significant differences (P < 0.001) between canine incidences over time.

Fig 4 shows the change in prevalence of dog seropositivity over time. From an initial overall seroprevalence of 24%, the seropositivity decreased significantly to 12% (*P* = 0.006) at 12 months before again, rising significantly to 35% (*P*< 0.001) at 24 months after intervention. As presented in Fig 5, the cumulative serological incidence for *L*. *infantum* infection among dogs also increased significantly from 8% to 32% (*P*< 0.001).

## Discussion

Although mass detection of infected dogs followed by culling was shown to have an impact in reducing the incidence of the canine and human VL [37], such approaches are often ineffective, costly and labour intensive, and difficult to implement from a public health perspective [17]. Hence, both the efficacy and acceptability of this control strategy are increasingly being contested [18]. Other approaches include the use of insecticide-impregnated dog collars [21] and fine-mesh impregnated bed-nets [38] but a sustainable prevention of the disease using these measures is costly and might fail in developing countries [22,23]. Nevertheless, an effective vaccine for helping at preventing progressive infection in dogs could provide long-term reductions in potential reservoirs that can limit transmission [24, 25].

Data from wide-scale field studies suggest an additive control effect of Leishmune^®^ vaccination over dog culling on the decline of the epidemics [39]. In this prospective field study, we analyzed the possible additive effect of Leish-tec^®^ vaccination over dog culling, on the decrease of infection and disease burden in a field population of naturally exposed dogs. Leish-tec^®^ vaccine is licensed only for individual prophylaxis and is recommended for healthy dogs. Nonetheless, the overall tolerance of the vaccine in dogs with either negative or positive serology for *L*. *infantum* infection appeared satisfactory in this study. The transient saponin-dependent toxic effects recorded in 11% of vaccine recipients is in agreement with the results obtained by other investigators [28].

Analysis of serum samples from Leish-tec^®^-vaccinated dogs indicated that the vaccine conferred humoral response against the rA2 protein without inducing cross-reacting antibodies to LPA or K28, the promastigote-derived antigens used as a marker of infection in the routine CanL immunodiagnostic tests. Comparable results were obtained in a previous study [36]. The fact that dogs seropositive to A2 were vaccinated raises the question of whether their antibody response was to vaccination or to natural infection. However, the levels of circulating anti-A2 IgG antibodies were significantly higher in vaccine recipients (either infected or non-infected dogs) than in infected control animals, thus confirming the immunogenicity of the rA2 antigen. Our data confirm the known high sensitivity (88%) of the A2-based ELISA for the detection of clinically asymptomatic canine carriers of *L*. *infantum* [35]. The high proportion (79%) of early-infected dogs disclosed by the A2-based ELISA corroborated previous findings, demonstrating that in areas of high transmission subclinical infections in dogs is widespread, involving as much as 63% to 80% of the population [40–42].

In this study, the anti-A2 IgG2 response was similar to that previously reported [36] in that a significant increase in IgG2 antibodies to A2 was observed throughout the tests in practically all tested vaccine recipients (*n* = 62). Of note, the 38 surviving vaccine recipients demonstrated IgG2, but not IgG1, antibody response to A2 at all-time points following immunization (data not shown) and remained healthy and K28- or LPA-seronegative by the end of the 2-year study. Interesting, this differential isotype humoral immune response was not generated in vaccine recipients unable to control *L*. *infantum* infection, thus supporting the view that monitoring these parameters in dogs could be an indirect way to distinguish serum samples of vaccinated or infected dogs in large-scale field studies [27].

Although antibody responses against sand fly saliva proteins are thought to limit initial parasite establishment [43], *Leishmania*-specific immunoglobulins apparently play no role in mediating protection and are often associated with disease exacerbation [9,25]. In this report, the levels of parasite-specific antibodies in diseased dogs were greater than those in sub-clinically infected ones, and a positive association was found between the dog’s anti-K28 antibody response, the clinical status and level of parasite burden as disclosed by histopathology analysis, which is in agreement with the results obtained by other researchers [14].

In a prospective study [28], 92.9% of Leish-tec^®^-immunized dogs remained healthy during the 11-month follow-up period. Studies evaluating Leishmune^®^ as a prophylactic vaccine in dogs exposed to the natural infection have shown similar rates of clinical outcome, ranging from 83.3% [44] to 95% [45]. It is interesting to note that by the end of this 24-month study, about a year after the last boosting vaccine dose, of the 62 vaccinated dogs that remained alive, 19 (31%) had been converted to a seropositive status only after five consecutive negative readings. In addition, the length of the periods devoid of an infection for vaccine recipients (mean seroconversion time of 18 months) was twice as high as that for sentinel dogs (mean seroconversion time of 9 months). These observations indicate that Leish-tec^®^-immunized animals more efficiently developed resistance mechanisms to eliminate leishmanial parasites once they invaded the host. However, dogs born resistant to *L*. *infantum* would also be able to maintain an effective cellular immune response against the parasite, and thus would not be infectious for sand flies. In a prospective field study of CanL [33], our estimates of serological reversion rates indicated at a high recovery rate among the identified seropositive canine population, suggesting that efficacious immune mechanisms exist in dogs living in households with natural exposure to the infection.

Previous studies in Brazil [10] showed that clinical status and parasite load of infected dogs are good markers of infectiousness and that a vaccine that prevented dogs becoming sick would reduce sand fly transmission by 97.5%. Therefore, control of VL by canine vaccination depends upon vaccine-induced protection against infectiousness to sandflies, rather than infection *per se*. conservatively, we assumed that vaccinated dogs would become infected, and that vaccine efficacy would be reflected in a decline in disease severity. Although a marked reduction in the rate of canine infection was seen when comparing vaccinated (27%) and control (42%) groups, protective responses were either not generated or not maintained in 43% of immunized dogs that became infected and developed disease. These results contrast with those achieved in the high dose *L*. *infantum*-beagle dog model, in which this rA2-saponine vaccine elicited protection against the onset of clinical VL [31]. The clinical outcome obtained in Leish-111f+MPL^®^-SE-vaccinated dogs naturally exposed to parasite transmission in a highly *L*. *infantum* endemic region in southern Italy [46] also stresses the striking differences that may exist between experimental and wide-scale field vaccine studies. Of 39 dogs enrolled in that study, 37 had been infected and developed the disease by the end of the 24-month study. The reasons for this are not clear but could be attributed to the presence of regulatory cytokines, notably IL-10 and TGF-β [13, 24]. One explanation is that sand fly transmission of parasites abrogates vaccine-induced protective immunity [47]. It is inferred that canines may receive up to one infectious bite/hour/night, under optimal vector conditions for transmission [47]. This may suggest that salivary gland antigens should be explored as a target for further vaccine development [25].

The projected additive effect of the preventive vaccination to the dog culling on the decrease of the incidence of CanL was not evident since comparable results were obtained previously during an intervention in the same study area attempting to remove potentially infectious dogs immediately upon detection [33]. The continued transmission observed in the intervention sites (as evidenced by the detection of newly infected animals every 6 months throughout the study) could be related to the incomplete elimination of infectious dogs, given that not all seropositive animals were culled during the study period. Furthermore, not all infected dogs were expected to be detected using serology, as the DPP CanL rapid test has a low sensitivity (47%) in identifying sub-clinically infected dogs [34]. The widespread infection among the dog population may also be related to the efficiency and timing of removing infected dogs and the effect of these measures in relation to seasonal variations in transmission rates of the parasite [10]. Of note, the number of seropositive dogs remarkably increased at the 24-month time point. It is worth mentioning that adult females of the local sand fly vector, *L*. *longipalpis*, are usually active from December through April, during which time the population density of these insects can become very high [15]. Congenital transmission of *L*. *infantum* was demonstrated in an experimentally infected beagle [48], but vertical transmission of the parasite from naturally infected dogs to offspring has not become evident [49].

One explanation for our results is that canines are not the primary reservoir for the maintenance of VL. If opossums serve as a paramount peridomestic reservoir of *L*. *infantum* [7, 8], the human disease may not be controlled in a community solely by culling infectious dogs [33]. Although humans naturally infected with *L*. *infantum* may play a role in transmission, it has been shown that clinically symptomatic, but not asymptomatic, cases are infectious to sandflies [50]. In this work, human-sand fly-canine transmission was unlikely, since no case of human VL was diagnosed during the study period.

In conclusion, Leish-tec^®^ showed promising protective effects but needs to be further optimized to be effective in dogs under field conditions. Our estimates also indicated that vaccination with Leish-tec^®^ in addition to dog culling program might not have an impact on the incidence of CanL in areas of high transmission. Finally, it should be noted that imperfect vaccines pose a threat because they are not completely sterilizing and allow more virulent strains to survive and transmit. If the evolved parasite strains then infected naïve, unvaccinated hosts, they will be more virulent than the strains that circulated before vaccine was used [51].

## Supporting information

S1 Table Changes in the K28-specific antibody levels in sera from Leish-tec^®^-vaccinated dogs shown by the duration of exposition to natural *L*. *infantum* infection. (DOCX)

S2 Table Changes in the K28-specific antibody levels in sera from control (sentinel) dogs shown by the duration of exposition to natural *L*. *infantum* infection. (DOCX)

S3 Table List of naturally exposed controls (sentinel) and vaccinated dogs that developed sub-patent or patent *L*. *infantum* infection during the 24-month period. (DOCX)
