# Supplementary material for: Field trial of efficacy of the Leish-tec® vaccine against canine leishmaniasis caused by Leishmania infantum in an endemic area with high transmission rates
Source: PLoS One. 2017 Sep 27;12(9):e0185438. doi: 10.1371/journal.pone.0185438 (PMC5617193; doi:10.1371/journal.pone.0185438)
Supplement: S1 Table — (DOCX) [file pone.0185438.s001.docx]

**S1 Table**. Changes in the K28-speciﬁc antibody levels in sera from Leish-tec®-vaccinated dogs*^a^* shown by the duration of exposition to natural *L. infantum* infection

|  | Antibody detection by the K28-based DPP kit*^b^* | | | | | |  |
| --- | --- | --- | --- | --- | --- | --- | --- |
|  | Time of follow-up (month) | | | | | | Seroconversion time (months) |
| **Dog code** | **0** | **4** | **6** | **12** | **18** | **24** | (mean = ̴ 18) |
| SLC01 | 0.0 | 0.0 | 0.7 | 0.2 | 1.0 | **22.3**^†^ | 24 |
| SLC03 | 0.0 | 0.3 | ND ⃰ | ND ⃰ | **36.9**^†^ | - | 18 |
| SLC05 | 0.5 | ND ⃰ | 0.4 | 0.0 | ND^§^ | - | - |
| SLC06 | 0.9 | 0.0 | 1.0 | 0.2 | 0.0 | ND^§^ | - |
| SLC07 | 0.1 | 1.9 | ND^§^ | - | - | - | - |
| SLC08 | 0.0 | 0.0 | 0.1 | 0.3 | 2.2 | ND^§^ | - |
| SLC10 | 0.6 | 0.3 | ND ⃰ | ND ⃰ | **68.9**^†^ | - | 18 |
| SLC11 | 1.9 | 0.4 | ND ⃰ | ND^§^ | - | - | - |
| SLC12 | 0.1 | 0.6 | 0.5 | 1.2 | **4.6** | ND ⃰ | 24 |
| SLC13 | 0.5 | 0.8 | ND^§^ | - | **-** | - | - |
| SLC14 | 1.0 | 1.5 | 0.8 | 1.7 | 0.0 | 1.8 | - |
| SLC15 | 0.2 | ND ⃰ | ND ⃰ | ND ⃰ | ND ⃰ | **6.0** | 24 |
| SLC17 | 0.1 | ND ⃰ | 0.0 | 0.0 | 2.3 | 2.2 | - |
| SLC20 | 0.1 | 0.0 | 0.4 | 0.0 | 0.8 | 1.9 | - |
| SLC24 | 0.0 | 0.6 | ND^§^ | - | - | - | - |
| SLC25 | 0.1 | 0.0 | 0.0 | 0.2 | **98.1**^†^ | - | 18 |
| SLC26 | 2.4 | 0.4 | 0.0 | ND ⃰ | ND^§^ | - | - |
| SLC27 | 0.3 | 0.1 | 0.4 | 0.3 | ND ⃰ | 0.5 | - |
| SLC28 | 0.0 | 0.0 | 0.1 | 2.7 | 0.4 | **5.9**^†^ | 24 |
| SLC29 | 0.4 | ND ⃰ | ND ⃰ | ND ⃰ | 1.1 | 0.1 | - |
| SLC31 | 0.1 | 0.4 | 0.5 | 0.0 | 1.4 | 0.6 | - |
| SLC32 | 1.0 | 0.0 | ND ⃰ | ND^§^ | - | - | - |
| SLC33 | 0.2 | 0.0 | ND^§^ | - | - | - | - |
| SLC34 | 0.0 | 1.5 | ND ⃰ | 1.1 | 0.4 | 1.4 | - |
| SLC36 | 1.1 | 0.9 | ND ⃰ | ND^§^ | - | - | - |
| SLC37 | 1.1 | 0.0 | ND ⃰ | ND^§^ | - | - | - |
| SLC38 | 0.2 | 0.0 | ND ⃰ | ND^§^ | - | - | - |
| SLC41 | 0.0 | 0.0 | 0.9 | 0.5 | 0.9 | ND^§^ | - |
| PC02 | 1.2 | ND ⃰ | ND ⃰ | **81.4**^†^ | - | - | 12 |
| PC03 | 0.3 | 0.0 | 0.6 | 0.4 | 0.3 | ND^§^ | - |
| PC04 | 0.4 | 0.2 | ND ⃰ | **98.6**^†^ | - | - | 12 |
| PC05 | 0.1 | ND ⃰ | ND ⃰ | **8.1** | ND^§^ | - | 12 |
| PC06 | 0.0 | 0.0 | ND ⃰ | **93.0**^†^ | - | - | 12 |
| PC07 | 1.3 | 2.9 | ND^§^ | - | - | - | - |
| PC08 | 0.4 | 2.6 | ND^§^ | - | - | - | - |
| PC09 | 0.1 | ND ⃰ | 0.0 | **31.6**^†^ | **-** | - | 12 |
| PC11 | 0.0 | 0.7 | 0.3 | 2.5 | ND ⃰ | **4.8**^†^ | 24 |
| PC13 | 0.1 | 0.1 | 0.5 | 0.0 | 0.4 | ND^§^ | - |
| PC14 | 1.4 | 0.0 | 0.2 | 1.0 | 0.0 | 0.5 | - |
| PC15 | 0.2 | 0.2 | 1.2 | ND^§^ | - | - | - |
| PC16 | 0.6 | 0.2 | 0.3 | 0.0 | 0.5 | ND^§^ | - |

**S1 Table.** (Continued)

| PC17 | 0.0 | 0.5 | ND ⃰ | ND^§^ | - | - | - |
| --- | --- | --- | --- | --- | --- | --- | --- |
| PC18 | 2.3 | 0.3 | 2.2 | 0.0 | 0.0 | 0.9 | - |
| PC19 | 0.0 | 0.3 | ND ⃰ | ND^§^ | - | - | - |
| PC20 | 0.1 | 0.0 | ND ⃰ | ND^§^ | - | - | - |
| PC21 | 0.8 | ND ⃰ | 0.4 | 0.0 | 0.4 | 1.4 | - |
| PC22 | 0.0 | ND ⃰ | 0.4 | 0.0 | 0.5 | 2.2 | - |
| PC23 | 0.0 | 0.1 | 1.5 | ND^§^ | - | - | - |
| PC24 | 0.3 | 0.0 | 1.5 | 0.5 | 1.2 | 2.5 | - |
| PC25 | 0.1 | 0.9 | ND^§^ | - | - | - | - |
| PC26 | 0.0 | 0.7 | ND^§^ | - | - | - | - |
| PC27 | 0.0 | ND ⃰ | 2.5 | ND ⃰ | ND ⃰ | **78.5**^†^ | 24 |
| PC28 | 0.6 | 0.0 | ND ⃰ | ND^§^ | - | - | - |
| PC34 | 2.5 | 2.3 | ND ⃰ | ND^§^ | - | - | - |
| RC01 | 0.2 | 0.0 | ND ⃰ | ND^§^ | - | - | - |
| RC02 | 0.0 | 0.0 | 1.2 | 0.0 | ND^§^ | - | - |
| RC03 | 0.5 | 0.0 | ND^§^ | - | - | - | - |
| RC04 | 0.0 | 0.0 | 0.0 | 0.2 | 0.0 | 0.3 | - |
| RC05 | 0.1 | 0.8 | 0.5 | 0.2 | 2.5 | **96.8**^†^ | 24 |
| RC08 | 0.1 | 0.7 | 0.2 | 0.7 | 0.2 | ND^§^ | - |
| RC10 | 1.0 | 0.0 | 1.5 | ND^§^ | - | - | - |
| RC13 | 1.5 | 0.5 | 2.8 | 2.8 | 0.4 | **19.8**^†^ | 24 |
| RC14 | 0.2 | 0.2 | 0.4 | 0.3 | 0.0 | 1.3 | - |
| RC16 | 2.4 | 0.0 | 0.3 | 0.7 | 0.4 | 0.5 | - |
| RC17 | 0.0 | 0.3 | 0.4 | 1.9 | 2.7 | **25.5**^†^ | 24 |
| RC18 | 0.5 | 0.9 | ND ⃰ | ND ⃰ | 0.6 | 0.9 | - |
| RC20 | 0.3 | 0.0 | 0.3 | 0.3 | **90.9**^†^ | - | 18 |
| RC21 | 0.2 | 0.0 | **4.0** | **56.6**^†^ | - | - | 6 |
| RC22 | 0.0 | 0.5 | 1.2 | 2.5 | 0.0 | ND^§^ | - |
| RC23 | 0.2 | 0.0 | 0.4 | 0.5 | 0.0 | 2.3 | - |
| RC24 | 0.1 | 0.7 | 0.0 | ND ⃰ | ND ⃰ | 0.0 | - |
| RC25 | 2.8 | 0.2 | 0.5 | 0.0 | 2.6 | 0.4 | - |
| RC26 | 0.2 | 0.0 | ND^§^ | - | - | - | - |
| RC27 | 0.2 | 0.6 | 0.6 | 0.0 | 2.5 | **16.6**^†^ | 24 |
| RC28 | 0.0 | 0.0 | 0.3 | 0.2 | 0.7 | **4.2** | 24 |
| RC29 | 0.2 | 0.2 | 0.7 | 0.1 | 0.3 | 2.7 | - |
| RC30 | 0.8 | 0.0 | 0.0 | 2.8 | **95.8**^†^ | - | 18 |
| RC31 | 0.1 | 0.0 | **4.8** | 0.3 | ND^§^ | - | 6 |
| RC32 | 0.6 | 0.1 | ND^§^ | - | - | - | - |
| RC33 | 0.0 | 0.0 | 0.1 | 0.2 | 2.0 | 0.8 | - |
| RC34 | 0.6 | 0.4 | ND^§^ | - | - | - | - |
| RC35 | 0.6 | 0.0 | 2.1 | 2.5 | **6.4** | **96.0**^†^ | 18 |
| RC36 | 0.0 | 0.5 | 0.5 | 0.0 | 2.3 | 1.8 | - |
| RC37 | 0.3 | 0.0 | 1.1 | 0.3 | 1.1 | 1.6 | - |
| RC38 | 0.0 | 0.0 | 1.8 | ND^§^ | - | - | - |
| RC39 | 0.0 | 0.0 | 0.6 | ND^§^ | - | - | - |

**S1 Table** (Continued)

| RC40 | 0.5 | 0.0 | 0.5 | 0.0 | 0.4 | **89.3**^†^ | 24 |
| --- | --- | --- | --- | --- | --- | --- | --- |
| RC41 | 0.0 | 0.3 | 0.0 | 0.0 | 0.3 | 0.1 | - |
| RC42 | 0.4 | 0.0 | 0.4 | 0.0 | 0.4 | **4.4** | 24 |
| RC43 | 0.0 | 0.0 | 1.0 | 0.4 | 0.8 | 0.6 | - |
| RC44 | 1.1 | ND ⃰ | 1.0 | 0.8 | 1.0 | 0.3 | - |
| RC46 | 0.0 | ND ⃰ | 1.3 | 0.5 | 2.1 | 1.0 | - |
| RC47 | 0.0 | 0.0 | ND ⃰ | ND ⃰ | ND ⃰ | 0.2 | - |
| RC48 | 1.1 | 0.3 | 1.9 | 0.8 | ND^§^ | - | - |
| RC49 | 0.0 | 0.4 | ND^§^ | - | - | - | - |
| RC50 | 0.2 | ND ⃰ | **6.8** | **26.9**^†^ | - | - | 6 |
| RC51 | 0.0 | ND ⃰ | 1.5 | 0.1 | ND ⃰ | 0.4 | - |
| RC52 | 0.0 | ND ⃰ | 0.9 | ND^§^ | - | - | - |
| RC54 | 2.5 | 0.8 | ND ⃰ | ND ⃰ | ND ⃰ | 2.9 | - |
| RC55 | 0.1 | 0.0 | 0.0 | 0.0 | 0.0 | 1.5 | - |
| RC56 | 0.5 | 0.0 | **81.8**^†^ | - | - | - | 6 |
| RC57 | 0.3 | 0.2 | 0.0 | ND^§^ | - | - | - |
| RC58 | 0.4 | 0.0 | 1.2 | 0.5 | 0.4 | 0.8 | - |
| RC60 | 0.0 | 0.0 | **4.2** | 0.5 | ND^§^ | - | 6 |
| RC61 | 0.2 | 0.2 | 0.4 | 0.2 | 0.4 | **4.8** | 24 |
| RC62 | 1.9 | 0.1 | **58.4**^†^ | - | - | - | 6 |
| RC63 | 0.5 | ND ⃰ | 0.7 | 1.0 | **46.8**^†^ | - | 18 |
| RC64 | 0.3 | ND ⃰ | 0.6 | 2.0 | 0.7 | **8.0**^†^ | 24 |
| RC66 | 0.4 | 2.2 | 0.3 | 0.7 | 0.3 | 0.9 | - |
| RC67 | 0.2 | 0.1 | 0.4 | 0.4 | 0.6 | 0.5 | - |
| RC68 | 0.1 | 1.5 | 0.8 | 0.6 | 1.7 | **40.6**^†^ | 24 |
| RC69 | 0.0 | 0.0 | 0.0 | ND^§^ | - | - | - |
| RC70 | 2.0 | 0.9 | ND^§^ | - | - | - | - |
| RC71 | 0.0 | 0.1 | 0.2 | 0.1 | ND^§^ | - | - |
| RC73 | 0.3 | 0.5 | ND^§^ | - | - | - | - |
| RC74 | 0.0 | 0.1 | 0.2 | 0.1 | 0.0 | 0.5 | - |
| RC75 | 0.3 | 0.0 | 0.1 | ND ⃰ | 0.4 | 0.0 | - |
| RC76 | 0.1 | 0.0 | 0.1 | 0.0 | ND^§^ | - | - |
| RC83 | 0.1 | ND ⃰ | 0.1 | 0.1 | ND^§^ | - | - |
| RC87 | 0.0 | ND ⃰ | 0.2 | 0.0 | **11.1**^†^ | - | 18 |
| RC93 | 0.0 | ND ⃰ | 2.6 | 0.0 | ND^§^ | - | - |
| RC94 | 0.1 | ND ⃰ | 0.2 | 0.5 | 0.0 | **95.5**^†^ | 24 |
| RC96 | 0.0 | 0.9 | ND^§^ | - | - | - | - |
| RC97 | 0.2 | ND ⃰ | 0.2 | 0.1 | 0.4 | 0.9 | - |
| RC98 | 0.6 | ND ⃰ | 0.2 | 0.0 | 0.9 | **95.5**^†^ | 24 |
| RC100 | 0.1 | ND ⃰ | 0.5 | 0.3 | 0.3 | 1.3 | - |
| UC01 | 0.0 | 0.1 | 1.0 | 0.2 | 0.1 | 1.5 | - |
| UC02 | 0.3 | 0.0 | 0.3 | 0.1 | ND^§^ | - | - |
| UC03 | 0.0 | 0.0 | ND^§^ | - | - | - | - |
| UC04 | 0.2 | 0.2 | ND^§^ | - | - | - | - |
| UC06 | 1.1 | 0.0 | 0.0 | 1.4 | 0.7 | 0.9 | - |

**S1 Table** (Continued)

| UC07 | 0.8 | 0.4 | ND^§^ | - | - | - | - |
| --- | --- | --- | --- | --- | --- | --- | --- |
| UC09 | 2.2 | 2.4 | ND^§^ | - | - | - | - |
| UC11 | 0.7 | 0.5 | 1.0 | 0.0 | ND^§^ | - | - |
| UC12 | 1.0 | 1.5 | ND^§^ | - | - | - | - |
| UC13 | 0.2 | 0.5 | ND^§^ | - | - | - | - |
| UC14 | 0.3 | 0.0 | 0.0 | ND^§^ | - | - | - |
| UC15 | 0.1 | 0.1 | 0.6 | 0.5 | 0.5 | 2.3 | - |
| UC16 | 0.0 | 0.1 | 0.2 | 0.2 | 0.1 | 0.1 | - |
| UC17 | 0.0 | 0.0 | 0.8 | 0.2 | ND^§^ | - | - |
| UC18 | 0.0 | 0.0 | 1.2 | 0.0 | 0.4 | 0.6 | - |
| UC20 | 0.0 | 0.1 | 0.0 | 1.4 | **11.8** | **87.8**^†^ | 18 |
| UC21 | 0.0 | 0.8 | 0.3 | 0.0 | 0.9 | 2.0 | - |
| UC23 | 0.3 | 0.9 | **17.7**^†^ | - | - | - | 6 |
| UC24 | 0.1 | 1.8 | ND^§^ | - | - | - | - |
| UC28 | 0.0 | 0.3 | 2.1 | **5.4** | ND^§^ | - | 12 |
| UC29 | 0.5 | 1.3 | ND^§^ | - | - | - | - |
| UC33 | 0.0 | 0.0 | 0.7 | 0.1 | 0.0 | ND^§^ | - |
| UC34 | 0.0 | ND ⃰ | ND ⃰ | 0.7 | 0.0 | ND^§^ | - |
| UC35 | 0.0 | ND ⃰ | 0.8 | ND^§^ | - | - | - |
| UC36 | 0.3 | 0.8 | ND^§^ | - | - | - | - |

*^a^*The K28-seronegative indigenous animals enrolled in the study were companion dogs (guard, hunting or “pet”) living in households with natural exposure to the infection.

*^b^* K28 specific antibody reactivity above the threshold of 3.0 RLU was considered as positive (note that all positive values appear in bold).

ND, not determined ( ⃰ At that time, animal could not be located for sampling; ^§^Animal died of other causes or relocated by the owner; ^†^Animal was euthanized 8 days after being diagnosed).
