# Supplementary material for: Field trial of efficacy of the Leish-tec® vaccine against canine leishmaniasis caused by Leishmania infantum in an endemic area with high transmission rates
Source: PLoS One. 2017 Sep 27;12(9):e0185438. doi: 10.1371/journal.pone.0185438 (PMC5617193; doi:10.1371/journal.pone.0185438)
Supplement: S2 Table — (DOCX) [file pone.0185438.s002.docx]

**S2 Table**. Changes in the K28-speciﬁc antibody levels in sera from control (sentinel) dogs *^a^* shown by the duration of exposition to natural *L. infantum* infection

|  | Antibody detection by the K28-based DPP kit*^b^* | | | | | |  |
| --- | --- | --- | --- | --- | --- | --- | --- |
|  | Time of follow-up (month) | | | | | | Seroconversion time (months) |
| **Dog code** | **0** | **4** | **6** | **12** | **18** | **24** | (mean = ̴ 9) |
| SLC35 | 0.1 | 0.7 | **12.7** | ND^§^ | - | **-** | 6 |
| SLC42 | 2.0 | 2.0 | **3.4** | ND^§^ | - | - | 6 |
| SLC43 | 0.0 | 0.3 | ND^§^ | - | - | - | - |
| SLC44 | 0.0 | 0.7 | ND^§^ | - | - | - | - |
| SLC50 | - | - | 0.5 | 0.9 | 0.4 | 1.8 | - |
| SLC56 | - | - | **-** | - | 0.4 | **3.5** | 6 |
| SLC57 | - | - | **-** | - | 0.0 | 0.6 | - |
| SLC58 | - | - | **-** | - | 1.4 | 0.4 | - |
| SLC59 | - | - | **-** | - | 0.1 | 2.8 | - |
| SLC60 | - | - | **-** | - | 0.2 | 1.6 | - |
| SLC65 | - | - | **-** | - | 0.7 | 1.7 | - |
| PC29 | 0.5 | 0.7 | ND^§^ | - | - | **-** | - |
| PC30 | - | 0.3 | 0.7 | ND^§^ | - | **-** | - |
| PC31 | 0.1 | 0.0 | **70.6**^†^ | - | - | **-** | 6 |
| PC32 | 0.0 | ND ⃰ | 1.5 | **33.4**^†^ | - | - | 12 |
| PC35 | 0.2 | ND ⃰ | 0.1 | 0.1 | 1.3 | 2.8 | - |
| PC38 | 0.0 | ND ⃰ | 0.0 | 0.1 | ND^§^ | - | - |
| PC39 | 0.2 | ND ⃰ | 0.3 | ND ⃰ | 0.2 | ND ⃰ | - |
| PC40 | - | 0.1 | ND ⃰ | ND ⃰ | 0.4 | ND^§^ | - |
| PC43 | - | - | - | 0.3 | 0.0 | **4.8** | 12 |
| PC44 | - | - | 0.2 | 0.5 | 0.4 | 0.4 | - |
| PC45 | - | **-** | 0.2 | **9.5** | **95.8**^†^ | - | 6 |
| PC46 | - | **-** | 0.3 | **23.4** | **96.2**^†^ | **-** | 6 |
| PC47 | - | - | 0.0 | 0.4 | 2.5 | ND ⃰ | - |
| PC48 | - | - | 0.7 | **6.8** | ND^§^ | - | 6 |
| PC49 | - | - | 1.5 | 0.0 | 0.3 | 0.5 | - |
| PC52 | - | 1.0 | **10.6** | ND^§^ |  |  | 2 |
| PC55 | - | - | - | 1.1 | 2.0 | 1.0 | - |
| PC56 | - | - | - | 0.6 | 2.8 | 1.1 | - |
| PC58 | - | - | - | - | 0.4 | 1.3 | - |
| RC102 | 0.9 | ND ⃰ | 0.4 | 0.0 | 0.2 | **3.6** | 24 |
| RC103 | - | - | 0.3 | 0.2 | 0.5 | **10.0** | 18 |
| RC104 | - | 0.7 | 0.6 | 0.0 | 1.0 | 1.1 | - |
| RC105 | - | 0.4 | 1.1 | 1.4 | ND^§^ | - | - |
| RC112 | - | - | 0.4 | 0.0 | ND^§^ | - | - |
| RC115 | - | - | 0.5 | 0.5 | 0.4 | 0.8 | - |
| RC116 | - | - | 0.6 | 0.0 | ND^§^ | - | - |
| RC117 | - | - | 0.6 | 0.0 | **7.1** | ND ⃰ | 12 |
| RC120 | - | - | 0.6 | 0.2 | 0.3 | **5.7** | 18 |
| RC121 | - | - | 0.6 | 0.2 | 0.6 | 1.0 | - |
| RC123 | - | 0.4 | **5.5** | ND ⃰ | ND ⃰ | **5.5** | 2 |

**S2 Table** (Continued)

| RC124 | - | - | 0.3 | ND ⃰ | 0.0 | 1.2 | - |
| --- | --- | --- | --- | --- | --- | --- | --- |
| RC125 | - | - | 0.0 | 0.0 | 0.3 | 0.5 | - |
| RC126 | - | - | 0.3 | 0.6 | ND^§^ | **-** | - |
| RC128 | - | - | **-** | 0.3 | 1.6 | **6.1** | 12 |
| RC129 | - | **-** | - | 0.1 | **7.9** | 1.8 | 6 |
| RC131 | - | - | - | 0.4 | 0.4 | 1.6 | - |
| RC132 | - | - | **-** | 0.3 | 1.0 | **5.0** | 12 |
| RC133 | - | - | - | **-** | 0.4 | **28.0**^†^ | 6 |
| RC134 | - | - | **-** | 1.0 | 0.0 | 1.0 | - |
| RC135 | - | - | - | 0.0 | 0.8 | 2.3 | - |
| RC136 | - | - | - | 0.4 | ND ⃰ | 2.6 | - |
| RC137 | - | - | - | 0.2 | 0.9 | 0.1 | - |
| RC138 | - | - | - | 0.2 | 0.0 | 1.2 | - |
| RC139 | - | - | **-** | 0.5 | 0.3 | **6.4** | 12 |
| RC141 | - | **-** | - | 0.3 | **3.7** | ND ⃰ | 6 |
| RC143 | - | - | 1.8 | **82.2**^†^ | - | - | 6 |
| RC144 | 0.9 | ND ⃰ | ND ⃰ | ND ⃰ | 0.6 | ND ⃰ | - |
| RC147 | - | - | **-** | - | 0.8 | **3.4** | 6 |
| RC150 | - | - | **-** | - | 0.6 | **5.4** | 6 |
| RC152 | - | - | **-** | - | 0.3 | **1.1** | - |
| RC155 | - | - | **-** | - | 0.1 | 0.8 | - |
| RC158 | - | - | **-** | 0.2 | **3.6** | ND ⃰ | 6 |
| RC159 | - | - | **-** | - | 2.0 | **96.5**^†^ | 6 |
| RC161 | - | - | **-** | - | 2.6 | **6.5** | 6 |
| RC162 | - | - | **-** | - | 2.0 | **5.9** | 6 |
| UC39 | 0.2 | ND ⃰ | 0.7 | **52.9**^†^ | - | - | 12 |
| UC41 | - | - | 0.2 | 0.0 | 0.1 | 0.2 | - |
| UC42 | - | - | 0.5 | ND ⃰ | 1.1 | ND^§^ | - |
| UC44 | - | - | - | 0.2 | 0.7 | 1.5 | - |
| UC46 | - | - | - | 0.0 | 1.0 | ND^§^ | - |
| UC47 | - | - | **-** | 0.1 | 0.8 | **14.7**^†^ | 12 |
| UC48 | - | - | **-** | 0.7 | 0.1 | 0.9 | - |
| UC49 | - | - | - | 0.0 | 0.2 | 0.6 | - |
| UC50 | - | - | 0.2 | **6.7** | ND^§^ | - | 6 |
| UC51 | - | - | - | 0.3 | 0.2 | ND ⃰ | - |
| UC54 | - | - | - | 0.0 | **99.8**^†^ | - | 6 |
| UC55 | - | - | **-** | - | 1.8 | **6.6** | 6 |

*^a^* The exposed unvaccinated group was composed of healthy K28-seronegative companion companion (guard, hunting or “pet”) dogs, including sentinel beagles^⁋^.

*^b^* K28 specific antibody reactivity above the threshold of 3.0 RLU was considered as positive (note that all positive values appear in bold).

ND, not determined ( ⃰ At that time, animal could not be located for sampling; ^§^Animal died of other causes or relocated by the owner; ^†^Animal was euthanized 8 days after being diagnosed).
