# Supplementary material for: Field trial of efficacy of the Leish-tec® vaccine against canine leishmaniasis caused by Leishmania infantum in an endemic area with high transmission rates
Source: PLoS One. 2017 Sep 27;12(9):e0185438. doi: 10.1371/journal.pone.0185438 (PMC5617193; doi:10.1371/journal.pone.0185438)
Supplement: S3 Table — (DOCX) [file pone.0185438.s003.docx]

**S3 Table**. List of naturally exposed controls (sentinel) and vaccinated dogs that developed sub-patent or patent *L. infantum* infection during the 24-month period

___________________________________________________________________________

Dog Animal Serological status determined by Clinical Parasite detected

codes group K28 DPP (RLU) LPA ELISA A2 ELISA form in affected tissues

| SLC42 | C | + (3.4) | ‒ (0.022) | + (0.309) | A | ND ⃰ |
| --- | --- | --- | --- | --- | --- | --- |
| RC147 | C | + (3.4) | ‒ (0.015) | + (0.446) | A | ND ⃰ |
| SLC56 | C | + (3.5) | ‒ (0.033) | + (0.408) | A | ND ⃰ |
| RC158 | C | + (3.6) | ‒ (0.024) | + (0.501) | A | ND ⃰ |
| RC102 | C | + (3.6) | ‒ (0.026) | + (0.770) | A | ND ⃰ |
| RC141 | C | + (3.7) | ND^§^ | ND^§^ | A | ND ⃰ |
| PC43 | C | + (4.8) | ‒ (0.030) | + (0.623) | A | ND ⃰ |
| RC132 | C | + (5.0) | ‒ (0.026) | + (0.308) | A | ND ⃰ |
| RC150 | C | + (5.4) | ‒ (0.015) | + (0.263) | A | ND ⃰ |
| RC123 | C | + (5.5) | ND^§^ | ND^§^ | A | ND ⃰ |
| RC120 | C | + (5.7) | ‒ (0.038) | + (0.734) | A | ND ⃰ |
| RC162 | C | + (5.9) | ‒ (0.036) | + (0.700) | A | ND ⃰ |
| RC128 | C | + (6.1) | ‒ (0.025) | + (0.860) | A | ND ⃰ |
| RC139 | C | + (6.4) | + (0.453) | + (2.033) | A | ND ⃰ |
| RC161 | C | + (6.5) | ‒ (0.027) | + (0.326) | A | ND ⃰ |
| UC55 | C | + (6.6) | ND^§^ | ND^§^ | A | ND ⃰ |
| UC50 | C | + (6.7) | ND^§^ | ND^§^ | A | ND ⃰ |
| PC48 | C | + (6.8) | + (0.354) | + (0.771) | A | ND ⃰ |
| RC117 | C | + (7.1) | + (0.453) | + (0.432) | A | ND ⃰ |
| RC129 | C | + (7.9) | ND^§^ | ND^§^ | A | ND ⃰ |
| RC103 | C | + (10.0) | + (0.338) | + (0.462) | A | ‒ |
| PC52 | C | + (10.6) | ND^§^ | ND^§^ | A | ND ⃰ |
| SLC35 | C | + (12.7) | ND^§^ | ND^§^ | A | ND ⃰ |
| UC47 | C | + (14.7) | + (0.386) | + (0.368) | A | ‒ |
| RC133 | C | + (28.0) | + (0.398) | + (0.308) | A | ‒ |
| PC32 | C | + (33.4) | + (0.433) | + (0.401) | A | ‒ |
| UC39 | C | + (52.9) | + (0.654) | + (0.568) | S | + |
| PC31 | C | + (70.6) | + (0.767) | + (0.468) | S | + |
| RC143 | C | + (82.2) | + (0.877) | + (0.428) | S | + |
| PC45 | C | + (95.8) | ND^†^ | ND^†^ | S | + |
| PC46 | C | + (96.2) | ND^†^ | ND^†^ | S | + |
| RC159 | C | + 96.5) | + (0.867) | + (1.162) | S | + |
| UC54 | C | + (99.8) | + (0.852) | + (1.933) | S | + |
| RC60 | V | + (4.2) | ND^§^ | ND^§^ | A | ND ⃰ |
| RC28 | V | + (4.2) | ‒ (0.150) | + (0.504) | A | ND ⃰ |
| RC42 | V | + (4.4) | ‒ (0.095) | + (0.666) | A | ND ⃰ |
| SLC12 | V | + (4.6) | + (0.462) | + (0.569) | A | ND ⃰ |
| PC11 | V | + (4.8) | ‒ (0.043) | + (1.280) | A | ND ⃰ |
| RC31 | V | + (4.8) | ‒ (0.087) | + (0.715) | A | ND ⃰ |
| RC61 | V | + (4.8) | ‒ (0.025) | + (1.381) | A | ND ⃰ |
| UC28 | V | + (5.4) | + (0.407) | + (0.834) | A | ND ⃰ |
| SLC28 | V | + (5.9) | + (0.142) | + (0.836) | A | ND ⃰ |
| SLC15 | V | + (6.0) | ND^§^ | ND^§^ | A | ND ⃰ |
| RC64 | V | + (8.0) | + (0.100) | + (1.099) | A | ND ⃰ |
| PC05 | V | + (8.1) | ND^§^ | ND^§^ | A | ND ⃰ |

**S3 Table** (continued) ___________________________________________________________________________

Dog Animal Serological status determined by Clinical Parasite detected

codes group K28 DPP (RLU) LPA ELISA A2 ELISA form in affected tissues

| RC87 | V | + (11.1) | ‒ (0.098) | + (0.890) | A | ND ⃰ |
| --- | --- | --- | --- | --- | --- | --- |
| RC27 | V | + (16.6) | ‒ (0.063) | + (0.549) | A | ‒ |
| UC23 | V | + (17.7) | + (0.312) | + (0.166) | A | ‒ |
| RC13 | V | + (19.8) | ‒ (0.330) | + (0.741) | A | ‒ |
| SLC01 | V | + (22.3) | + (0.245) | + (1.479) | A | ‒ |
| RC17 | V | + (25.5) | ‒ (0.101) | + (0.615) | A | ‒ |
| RC50 | V | + (26.9) | ND^†^ | ND^†^ | A | ‒ |
| PC09 | V | + (31.6) | ND^†^ | ND^†^ | A | ‒ |
| SLC03 | V | + (36.9) | + (0.500) | + (0.928) | A | ‒ |
| RC68 | V | + (40.6) | + (0.181) | + (1.032) | A | + |
| RC63 | V | + (46.8) | ‒ (0.053) | + (2.936) | S | + |
| RC21 | V | + (56.6) | ND^†^ | ND^†^ | S | + |
| RC62 | V | + (58.4) | ND^†^ | ND^†^ | S | + |
| SLC10 | V | + (68.9) | + (0.229) | + (0.559) | S | + |
| PC27 | V | + (78.5) | + (0.447) | + (0.556) | S | + |
| PC02 | V | + (81.4) | + (0.654) | + (0.846) | S | + |
| RC56 | V | + (81.8) | ND^†^ | ND^†^ | S | + |
| UC20 | V | + (87.8) | + (0.660) | + (1.634) | S | + |
| RC40 | V | + (89.3) | + (0.271) | + (0.710) | S | + |
| RC20 | V | + (90.9) | + (0.601) | + (0.848) | S | + |
| PC06 | V | + (93.0) | + (0.766) | + (0.736) | S | + |
| RC94 | V | + (95.5) | + (0.268) | + (1.090) | S | + |
| RC98 | V | + (95.5) | + (0.528) | + (1.297) | S | + |
| RC30 | V | + (95.8) | + (0.400) | + (0.769) | S | + |
| RC35 | V | + (96.0) | ND^†^ | ND^†^ | S | + |
| RC05 | V | + (96.8) | + (0.576) | + (1.042) | S | + |
| SLC25 | V | + (98.1) | + (1.080) | + (1.758) | S | + |
| PC04 | V | + (98.6) | + (0.314) | + (0.511) | S | + |

Animals were assessed through time by the rapid rK28 fusion protein chromatographic immunoassay to measure seropositivity for *L. infantum* infection. The test procedure was that as described previously [35]. K28 specific antibody reactivity above the threshold of 3.0 RLU was considered as positive. Results using LPA-based ELISA kits were evaluated with the instructions provided by the manufacturer as described in the Methods section. ELISA to measure anti-A2 total IgG antibodies was performed using the subunit A2-HIS protein from *L. donovani*, prepared as previously described [32]. The effect of the vaccination on pathological changes in necropsy tissues was evaluated as previously reported [33].

C, control; V, vaccinated; A, asymptomatic; S, symptomatic.

^§^ Not determined (animal could not be located or owner refused serum sampling).

^†^ Dog was necropsied but serum sampling was not performed at that time.

⃰ Necropsy was not performed in this animal.
